# Control and Supervision Requirements for Floating Hybrid Generator Systems

**DOI:** 10.3390/ijerph191912781

**Published:** 2022-10-06

**Authors:** Emilio García, Antonio Correcher, Eduardo Quiles, Fernando Tamarit, Francisco Morant

**Affiliations:** Instituto de Automática e Informática Industrial, Universitat Politècnica de València, Camino de Vera, s/n, 46022 Valencia, Spain

**Keywords:** renewable energy, marine energy, floating wind generators, marine current turbines, tidal turbines, wave energy converters, supervisory control engineering, condition monitoring

## Abstract

This work presents a series of devices that generate renewable energy from the marine environment which, in recent years, have aroused increasing interest. In particular, the main types of floating wind generators and marine current turbines are described. Over time, some of these floating generators have evolved in various hybrid modalities, integrating different generation devices into the same system, wind turbines, marine current turbines, wave energy converters, etc., with the objective of multiplying their generation capacity and optimizing the investment made in the floating system. However, this hybridization offers, in some cases, an opportunity to address the problem of controlling the structural stability of the system. Such stability enhancement has been considered a major challenge since the early days of floating wind turbine design. With this objective, in this work, a specific solution is proposed, consisting of a floating hybrid system composed of a wind generation subsystem and a generation subsystem with two marine current turbines. This proposal allows the development of an integrated control system which deals simultaneously with the structural stability of the system and the optimization of the generation capacity. Additionally, other requirements are also highlighted relating to the achievement of economic viability objectives, considering the reliability and availability of the system in the particularly aggressive marine environment, where maintenance operations are especially costly. In this sense, a model of intelligent integration of the tasks of supervision, diagnosis, and predictive maintenance is proposed.

## 1. Introduction

The European Union (EU) revised its Renewable Energy Directive (2009/28/EC) in May 2022, aiming for 45% of energy consumed to be renewable energy by 2030. This is a new step towards achieving the goal of climate neutrality by 2050.

This roadmap requires increasing wind power and power from other renewable sources, which environmental and land use reasons may condition. Marine energy is still at an early stage compared to the rest of the renewable energy sources, but its potential is very high. The installation of offshore wind farms is an option that would increase the installed wind power, avoiding some of the drawbacks of conventional wind farms, especially the visual impact and the impact on avian fauna. At the end of 2019 [1], the global installed power of offshore wind farms was 23 GW (80% in Europe), which accounted for 0.3% of global electricity generation. Most of these wind farms are installed in Northern Europe [2]. Denmark has the largest installed capacity, with 2.97 GW in 2019 and 8 GW more under development. In addition, China has started installing this technology, with 1.23 GW installed and 1.4 GW under construction.

Globally, installing offshore wind farms is seen as one of the areas with the most significant potential for future development within the wind energy sector. Nevertheless, there are other marine energy power generators to be considered.

Floating wind turbines (FWTs) [3,4] are the alternative to offshore wind power generation for depths greater than 60 m. Operating at a distance from coasts involves using more important wind resources and reducing the visual and noise impact. Nevertheless, the floating nature of these devices involves stability requirements for the control system in addition to the power production requirements.

Marine currents [3] stand out among the possible sources of energy from the sea, the theoretical foundations of which are very similar to those of wind energy. Marine current turbines [4] (MCTs) use the kinetic energy of marine currents to obtain power in a similar way to how wind turbines (WTs) use wind. The maximum use of the necessary marine installations allows consideration of the integrated installation of other generating devices such as MCTs, which are experiencing development and have a promising future. The aspect that is being worked on the most is the generator control design. Turbines are usually coupled with permanent magnet synchronous generators that have nonlinear behavior. Thus, the control strategy that is applied has a substantial impact on the energy that the turbine can capture. Recent research on advanced nonlinear controls [5,6] reported simulation results that increased the extracted energy by 20% compared to classical control techniques and faster transient responses.

Wave energy can be, in the future, a significant alternative to fossil fuels. It is estimated that the use of wave energy will increase significantly over the next few decades [7,8,9,10,11]. Wave energy converter (WEC) technology is still in the development phase, with numerous pilot studies going on around the world [12,13,14,15]. According to a European Technology and Innovation Platform for Ocean Energy study published in May 2020 [13], by 2050, ocean energy could deliver 100 GW of capacity, equivalent to 10% of Europe’s electricity consumption today.

Using multiple energy sources simultaneously in the form of microgrids is common practice with conventional renewable energy sources such as solar and wind. These sources are often integrated with storage systems to facilitate their connection to the grid. Marine energy research is also trying to integrate multiple sources simultaneously. A recent example is the work in [16], which presented a simulated microgrid model that integrates batteries, photovoltaic panels, tidal turbines, and wind turbines.

Another example of hybridization is the joint use of wind turbines and MCTs on floating platforms. Hybrid FWT and MCT foundations [17] are promising generators that can achieve more excellent stability than FWTs. These foundations are a hot topic in the research and study of control algorithms and marine power generation.

The traditional objective of control systems applied to hybrid generation systems has been to maximize the energy generated. In the case of generation systems resting on a floating offshore platform, other requirements may be equally or more important. Due to the difficulty of access, costly maintenance, and expensive commissioning involved in having generators offshore, ensuring the physical integrity of the generator is the priority. Since offshore environmental conditions can be extreme, controllers and monitoring systems must prioritize platform stability. Different generation systems working on the same platform complicate system operation but present many opportunities for controller design strategies to ensure platform stability.

This work proposes a specific solution consisting of a floating hybrid system composed of a wind generation subsystem and a generation subsystem with two marine current turbines. This proposal allows the development of an integrated control system, which deals simultaneously with the structural stability of the system and the optimization of the generation capacity.

The novelty of this proposal is that it introduces the concept of cooperative, integrated control of the two generation subsystems involved to counteract tendencies towards instability, which, if not avoided, reduce the useful life of the hybrid system. This issue is highlighted as especially important in [18]. This is an inherent problem with hybrid floating systems where, under certain circumstances, the applied pitch control can cause the floating system to resonate.

This paper is organized as follows: Section 2 reviews the main FWT foundations. Section 3 discusses wind turbine control in FWT devices. Section 4 focuses on marine current turbine technologies. The hybrid (FCT and MCT) concept is presented in Section 5. Section 6 discusses the requirements for the supervision system for all these technologies. Finally, Section 7 presents the conclusions.

## 2. Floating Offshore Wind Turbines

For some years now, the convenience of moving wind farms to the marine environment has been considered to achieve more favorable conditions for power generation. Relating to this, several projects have been developed worldwide to test the feasibility of floating FWTs.

The first prototype [19], Blue H, was installed in 2008 in a water depth of 113 m. Since then, researchers and firms have commissioned other prototypes.

Windfloat^®^ is a patented FWT developed by Principle Power Inc. (Priciple Power Inc., Emeryville, CA, USA). The Windplus consortium used this WT to design the first floating wind farm. The first turbine was installed in 2010, and, in July 2020, the floating wind farm was fully operational. WindFloat Atlantic is grid-connected to Portugal, generating up to 25 MW.

RWE global is also working on FWT prototypes. DemoSATH (Saitec Offshore Technologies, IngZero, and Fundación Instituto de Hidráulica Ambiental de Cantabria, Leioa, Spain) is a 2 MW turbine over a concrete structure. The prototype has a single point of mooring that provides self-alignment to the current and wave direction. This project is installed on the north coast of Spain. TetraSpar (Stiesdal, Copenhagen, Denmark) is a 3.6 MW turbine over a tubular steel structure. The prototype test site is located in Denmark, 10 km offshore with a depth of 200 m. Aqua Ventus (Cianbro Corporation and University of Maine, Orono, ME, USA) is an 11 MW WT over a concrete, semi-submersible structure. This project is expected to be operative in 2024 in New England (USA).

A disrupting FWT system is being developed by X1wind (X1wind, Barcelona, Spain). This firm has developed PivotBuoy^®^, a system capable of self-orientating the floating turbine to maximize the generated power, thus, reducing the weights and making FWTs more competitive.

Real prototypes and scientific research [20,21] show that the control of FWTs faces challenges that cannot be overcome with conventional power control techniques. High structural loads or platform movement due to wave and tidal currents impose stability restrictions not considered in traditional WT control. This vital requirement points to the need for more advanced predictive control and condition monitoring techniques than those in conventional power control to preserve structural stability and integrity.

### 2.1. Mooring Systems

Supposing the FWT is installed with a depth higher than 50 m, in this case [22], it is necessary, for economic viability reasons, to opt for floating structures as opposed to supported alternatives and anchoring to the seabed. This additional condition increases the system’s complexity, considering the hydrodynamic stability conditions of a floating system with six degrees of freedom [23].

**Figure 1:**
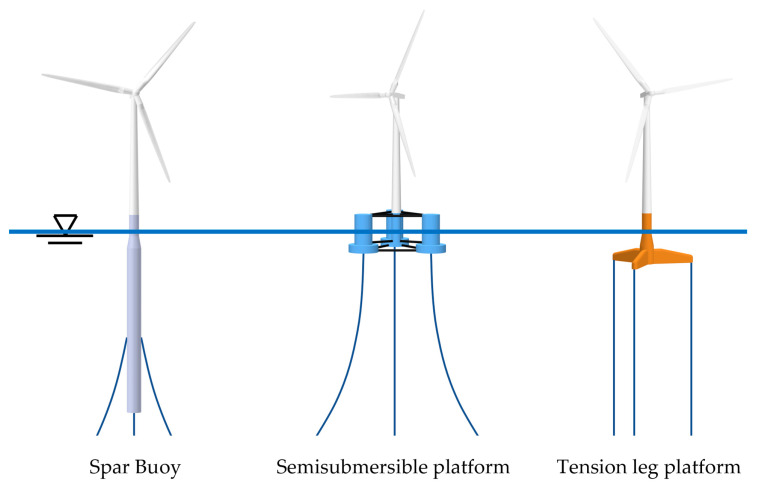
FWT mooring systems.

To this end, experts have considered various mooring systems [19,24]. There are many mooring concepts, but they can be categorized into three types. Figure 1 illustrates these categories.

The spar buoy concept achieves stability through the use of ballast to bring the center of gravity (CoG) below the center of buoyancy (CoB) and can be moored by catenaries or tension lines [25]. The tension leg platform (TLP) achieves stability by using mooring tension lines caused by excessive buoyancy in the tank [26]. Finally, the semi-submersible concept achieves stability partly with the ballast and the floater [27]. Other hybrid ideas have also been developed using the characteristics of the three classes described in [19].

#### 2.1.1. Spar Buoy

The design of the spar buoy is based on keeping the center of gravity below the center of buoyancy, using a ballast located below the sea surface, thus, trying to achieve stability. It can be moored to the seabed using catenaries or tensioning lines.

This design was first used in the Hywind prototype developed in 2009 [24], which included a 6-MW-scale WT. Dynamically, the turbine behaves as a nonlinear mass-spring-damper system. It is excited by hydrodynamic forces from waves and currents and wind-induced forces. The mooring system consists of several anchors (usually three) embedded in the seabed. When the turbine experiences a thrust force from a single anchor, the line holds and acts as a spring, thus, pulling the turbine back into position. Damping is provided primarily by hydrodynamic forces.

#### 2.1.2. Tension Leg Platform

The platform is permanently tied down using vertical tendons that are grouped at each corner of the structure. A feature of the tie-down design is that it has relatively high axial stiffness (low elasticity) so that virtually all vertical movements of the platform are eliminated.

Examples of this type of mooring are [28] GICON-SOF (Gicon), Eco TLP, or TLPWind (Iberdrola).

#### 2.1.3. Semi-Submersible Platforms

This kind of foundation includes a series of columns linked together with tubular, light structures. The wind turbine is typically placed on one of the columns, but some designs place the turbine in the geometric center. The whole structure is partly submerged, which makes it versatile for a wide range of depths. An example of this kind of FWT is WindFloat by Principle Power Inc.

Research on the dynamics of these platforms [29] has shown that TLPs are more flexible in sway but hard in rotational modes. Semi-submersible platforms are flexible and easy to develop. Therefore, it is the first design option for complex FWT prototypes.

### 2.2. Structural Loads Considerations

In a conventional wind turbine with structural support to the ground, the angular displacement of the tower is comparatively small, even under challenging wind conditions. The axial force of the wind mainly causes bending moments in the tower. Under these conditions, the weight of the nacelle acts by compressing the tower not bending it.

In an FWT, the support platform moves freely, and the tower can experience angular displacements of several degrees. In this case, the weight of the nacelle is directly related to the bending of the tower. Of note in this phenomenon are the effects of its amplitude and frequency. In terms of frequency, due to constantly varying wind and wave loads, significant fatigue stresses occur in the structure.

These considerations lead to the subjection of special operating conditions worthy of detailed analysis, which can only be performed with the help of simulation software tools. Specifically, the simulation tool FAST [30] is highlighted for this purpose.

In addition, to perform such analysis, it is necessary to have a coupled dynamic behavior model of the floating support base and the tower/nacelle. This model helps to define which variables should be monitored in the context of a condition monitoring system of the FWT structural system.

Everything points out that attention must be paid to the interactions between the mechanical effects due to inertia loads (rotor, nacelle, and tower) and the electrical effects (generator, control, and protection systems).

### 2.3. Implications of the FWT Control Design on Structural Loading

From the control engineer’s point of view, an FWT is a system with low actuation capability. The main control inputs are the rotor blade angle (pitch) and the nacelle’s yaw angle (yaw). Depending on the generator technology, active generator torque control is also available. In principle, no actuators can actively control the position and orientation of the platform itself.

Little can be done about rotational and transverse movements. The control must rely on the mooring system and the hydrostatic and hydrodynamic forces doing their job. On the contrary, the controller can command the axial thrust of the wind turbine, as this can influence the tilting motion of the FWT.

Wave movements and variations in wind speed can cause turbine movements. Ideally, it is desirable to keep the wind turbine as structurally stable as possible for the blade swing angle control. At the same time, control actions producing negative damping (or, equivalently, increases in the amplitude of the FWT swinging motion) should be avoided. For example, when using a pitch control design to reduce the rotor air resistance as the nacelle moves forward, negative damping is produced [18].

Conventionally, wind turbines alternate between two types of pitch control action [31]:

- When the wind speed is below the nominal wind speed, the pitch control tries to maximize the power output, keeping the tip speed at an optimal ratio;
- Above the nominal wind speed, the rotor blades are angled to maintain turbine operation at a constant speed and torque.

In modern wind turbines, this is performed in two different ways. The first is collective pitch control. This control strategy is widely implemented in commercial wind turbines, and its principal characteristic is that the pitch reference is set collectively for all the blades. The control method is generally a PID-based algorithm, although there is research on more complex algorithms such as the sliding control [32] or fuzzy logic [33] algorithms. The second is individual pitch control. This strategy has been researched in the last ten years, and it is in the process of development for commercial turbines [34].

To ensure minimum mechanical wear of the turbine components, the concept of constant power output may have to be sacrificed, and the regulation of power variations be performed on the ground, using, for example, flywheel energy storage techniques [35]. The blade angle can be used to control the turbine’s axial thrust, which can dampen the pitch movement. The generator control must adopt torque variations, and this must be performed to reduce wear on the blade roots, rotor bushing, and gearbox shaft. These strategies require generator technology that allows us to control the torque, which limits the design to fed converter designs such as impulse-direct PMSM design, doubly fed induction generators (DFIG), or induction generators with a full power converter.

The controller’s goal should be to minimize turbine and platform motion while limiting mechanical wear in the generator and transmission. In this regard, some simulation tools, such as FAST [36], have system linearization tools that can be used to design controllers based on linear–quadratic gain theory. Another option is the design of controllers based on Lyapunov theory for the minimization of energy functions. The FAST simulation tool was specifically developed to carry out pre-study tests on the overloads that can occur, among other things, on the blades and tower of the floating wind turbines. IT allows the testing of different control strategies, such as gain scheduling PID, LQR with collective blade pitch, and LQR with individual blade pitch or H∞.

## 3. Wind Turbine Control

For onshore wind farms, the main objective of conventional, active control techniques is to regulate the power generated [31]. This is achieved by appropriately varying the blades’ angle of attack in opposition to the wind. Control strategies vary depending on whether the wind speed is above or below the rated value.

- When the wind is too low (region 1), the generated power does not compensate for the losses on the mechanical part, so the WT is stopped;
- The WT can generate power under its rated value between the cut-in and the nominal wind speed (region 2);
- Region 3 is reached at rated wind speed, thus, producing the rated power;
- When the wind reaches high regimes over the nominal speed, the WT is switched off for safety reasons.

In region 3, the active pitch control maintains the rotor speed constant by varying the pitch angle. A change in the angle modifies the wind power input to the turbine, thus, changing the rotor speed.

The WT power generation efficiency can be modeled as:

(1) $$P_{w}=C_{p}\left(\lambda ,\theta \right)\frac{1}{2}\;\rho \;A\;u^{3}=C_{p}P_{v}$$

where *P_w_* is the harnessed power, *P_v_* is the power contained in the wind, *C_p_* is the power coefficient, r is the air density, A is the area swept by the rotor, and u is the wind speed. The power coefficient is not constant but varies with the tip speed ratio (*λ*) and blade parameters such as the pitch angle (*θ*). However, the constant increase in rotor diameter to increase the WT generating capacity involves higher structural loads being borne by the WT.

More precisely, the dynamics of large, horizontal-axis wind turbines can be modeled using a five-degrees-of-freedom model. The dominant modes [18,37] include:

- Out-of-plane deflection of the blade flap rotor;
- In-plane deflection of the blade edge;
- Fore and aft tower motions;
- Powertrain roll and twist.

**Figure 2:**
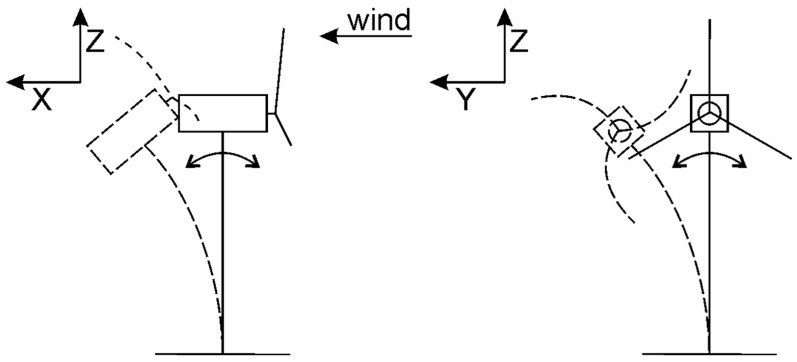
DoF of a WT.

As suggested in Figure 2, the dynamics of the deformation associated with these degrees of freedom tend to be coupled.

For example, the tower fore–aft motion is strongly coupled to the blade flap motion, and the tower roll motion is strongly coupled to the blade edge and powertrain torsion.

In this context, large, modern WTs allow the application of control techniques that make possible an independent adjustment of the pitch angles of each blade [31]. Individual pitch control extends the conventional objectives of pitch control to include reducing fatigue loads, particularly by the active damping of tower oscillations.

In the case of complex floating systems with six degrees of freedom, the system behaves as a mass-spring-damper system affected by changing forces resulting from wind flows and hydrodynamic forces due to waves and sea currents. Under certain conditions of higher-than-normal wind speed, conventional pitch control techniques introduce negative damping in the movement of the floating tower. This causes an excitation of the natural frequency and may cause the floating structure to resonate by applying decreases in the wind opposition when varying the pitch angle of the blades to regulate the active power generated. This phenomenon was observed in tests carried out at the Ocean Basin Laboratory at Marintek in Trondheim [38].

The following six coordinates describe the motion of a system with six degrees of freedom:

(2) $$q=\left[\begin{array}{ccc} x & y & z \end{array}\;\begin{array}{ccc} \Phi & \Theta & \Psi \end{array}\right]$$

**Figure 3:**
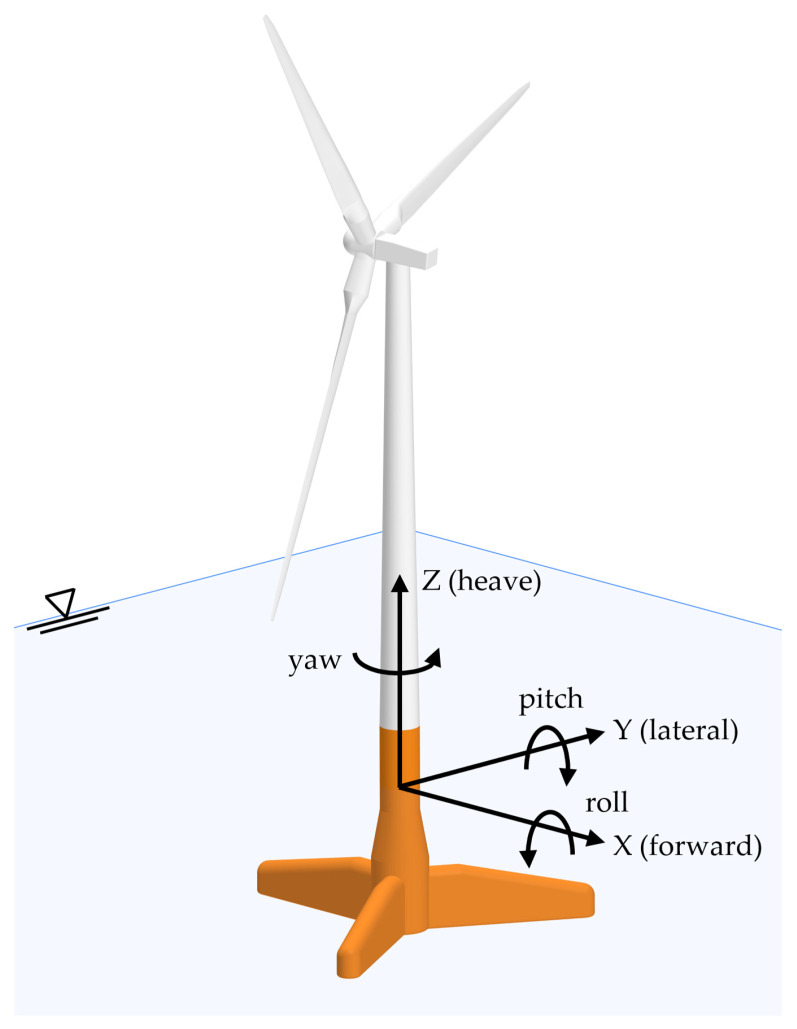
DoF of an FWT.

In Equation (2), six independent coordinates are used. The first three describe the translational motions on the *x* (forward), *y* (lateral displacement), and *z* (heave) axes. The last three coordinates represent the rotational motions Φ, Θ, and Ψ, called the roll, pitch, and yaw (Figure 3). On the other hand, the equation of motion of a system possessing j degrees of freedom, moving around a stationary point in a fluid, is:

(3) $$\sum_{k=1}^{6}m_{jk}{\ddot{q}}_{k}=\tau_{j}^{H}+\tau_{j}^{R}+\tau_{j}^{D}+\tau_{j}^{A}+\tau_{j}^{E}$$

where *q_k_* is the k-th coordinate of the body, *m_jk_* is the mass and inertia parameters, $\tau_{j}^{H}$ is the hydrostatic forces, $\tau_{j}^{R}$ is the radiation forces in the form of waves due to the body’s motion, $\tau_{j}^{D}$ is the diffraction forces due to waves breaking against the body, $\tau_{j}^{A}$ is the acting forces, and $\tau_{j}^{E}$ is the other external forces.

The terms added mass and potential damping typically model the radiation forces. The added mass is often mistakenly taken to represent an amount of water that is “fixed” to the structure and moves with it. It is simply a practical representation of the hydrodynamic forces that are proportional to the acceleration of the body. The hydrostatic forces can be represented as a restoring force proportional to the deviation from neutral. Then, the equation of motion is commonly written as:

(4) $$\sum_{k=1}^{6}(m_{jk}+\alpha_{jk}\left(\omega \right)){\ddot{q}}_{k}+\sum_{k=1}^{6}\beta_{jk}\left(\omega \right)\dot{q_{k}}+\sum_{k=1}^{6}c_{jk}q_{k}=\tau_{j}^{D}+\tau_{j}^{A}+\tau_{j}^{E}$$

where *α_jk_*(*ω*) is the frequency-dependent added mass, *β_jk_*(*ω*) is the frequency-dependent damping potential, and *c_jk_* is the restoration coefficient.

The added mass coefficients and damping potentials are commonly found from experiments or by software such as WAMIT, which calculates estimates.

Radiation forces can be represented with convolution integrals:

(5) $$\overset{6}{\underset{k=1}{\sum }}(m_{jk}+\alpha_{jk}){\ddot{q}}_{k}+\overset{6}{\underset{k=1}{\sum }}b_{jk}\dot{q_{k}}+\overset{6}{\underset{k=1}{\sum }}c_{jk}q_{k}+\overset{6}{\underset{k=1}{\sum }}\int_{-\infty }^{t}K_{jk}\left(t-\sigma \right){\dot{q}}_{k}\left(\sigma \right)d\sigma =\tau_{j}^{D}+\tau_{j}^{A}+\tau_{j}^{E}$$

where *K_jk_*(*t*) can be viewed as an impulse response function in direction *j* at an impulse velocity in direction *k*. The convolution integral of (5) is usually represented as:

(6) $$\mu_{jk}=\int_{-\infty }^{t}K_{jk}\left(t-\sigma \right){\dot{q}}_{k}\left(\sigma \right)d\sigma$$

However, instead of the convolution integral, the following linear system is usually used because it is simpler to implement and solve in software simulation systems. Additionally, the execution of the simulations is faster.

(7) $${\dot{\xi }}_{jk}=A_{jk}\xi_{jk}+B_{jk}\dot{q_{k}}$$

(8) $$\mu_{jk}=C_{jk}\xi_{jk}+D_{jk}\dot{q_{k}}$$

The coefficients are obtained from the Laplace transform of the impulse response function.

*K_jk_*(*s*) = **C**(*s*I − **A**)^−1^ + **D** (9)

From a comparative point of view, the control requirements of WT and FWT have no differences. In both, the desired objective is power regulation to maximize energy capture. When the wind speed exceeds the nominal value, the primary objective is to minimize structural fatigue due to overloads.

In the case of the FWT, the latter objective is of greater importance since it is a six-DoF system exposed to more abrupt movements due to the superimposed effects of wind and sea. Additionally, each of the three types of floating platform (Figure 1) introduces different static and dynamic characteristics that respond differently to the same control strategy.

Studies were initially conducted with individual target controllers for rotor speed regulation using collective blade pitch [39] for different mooring systems [40]. The first study [41] performed extensive analyses with all three platforms using a gain-programmed proportional integral controller. Their results showed that the barge platform had the highest tower and blade loads and the most significant movements in the wind turbine platform. The TLP was the best in this respect. In the last ten years, individual pitch control has been the main trend [20]. In [42], an individual pitch control scheme was developed to deal with blade and pitch actuator faults in FWTs. It is shown that, under these faults, conventional pitch control techniques fail. Modern techniques [32] (such as sliding control) have also been applied to pitch control in an FWT, with promising results. The proposed controllers can accomplish better power regulation, reducing the platform pitch motion and the blade load.

## 4. Power Generation through Marine Current Turbines

Exploiting ocean currents has been considered a realistic energy supply option due to recent improvements in offshore engineering technology [43]. Ocean currents deserve careful consideration, as they have the potential to supply a significant fraction of European future electricity needs and could enable the development of a major industry to produce clean energy for the 21st century [44].

Recent research [3] showed a potential of 26,000 TWh every year, including both tidal current and tide energy. Although this resource appears to have great potential as a renewable energy source, it has so far been neglected as an area of research.

These systems use the kinetic energy of water movement to obtain electrical power from turbines similar to WTs, so-called flow turbines. This technology is gaining popularity due to its lower cost and ecological impact compared to tidal power plants that use dams to generate potential energy. In the latter, the civil work associated with dam construction requires high civil infrastructure costs; there is a worldwide shortage of viable sites for operation, and their environmental impact can be high.

Modern advances in turbine technology make it possible to obtain large amounts of power generated from the oceans using the flow of ocean currents. Some turbines can be arranged in areas where high-velocity ocean currents naturally exist due to the concentration of current flows, such as on Canada’s west and east coasts and in the Strait of Gibraltar, the Bosphorus, and numerous sites in Southeast Asia and Australia. Such currents occur almost everywhere: entrances to bays and rivers or in narrowings between land masses where water currents are especially concentrated.

### 4.1. Marine Current Turbines

For the energetic exploitation of marine currents, different models of turbines have been designed to take advantage of the kinetic energy of these currents.

The kinetic energy of these systems can be expressed as:

(10) $$P=C_{p\;}\cdot 0.5\;\cdot \;\rho \;\cdot A\;\cdot V^{3}$$

where *P* is the generated power, *C_p_* is the turbine power coefficient, *r* is the density of water (in seawater, it is approx. 1025 kg/m^3^), *A* is the turbine’s swept area, and *V* is the flow velocity.

Harnessing the energy in a tidal flow requires converting the kinetic energy of a moving fluid, in this case, water, into the motion of a mechanical system that drives a generator. It is not surprising that many developers have concurred in suggesting the use of technology that mirrors what has been successfully used to harness wind, which is also a moving fluid. In addition, much of the technology is based on horizontal-axis turbines, such as the one shown in Figure 3. However, there are several differences in the design and operation of offshore turbines. Distinct differences involve changes in force loads, submergence, stall mode characteristics (hydrodynamic versus aerodynamic stall input), and, above all, the specific characteristic of marine current turbines (MCTs) in eventual cavitation.

Turbine rotor aerodynamics refers to the interaction of the wind turbine rotor with the incoming wind. The treatment of rotor aerodynamics in all current design codes is based on the well-known and well-established Glauert method of blade element momentum (BEM) theory. The BEM method has therefore also been used for rotor modeling of marine turbines. Indeed, it is widely used in the industry as a computational tool to predict the aerodynamic/hydrodynamic loads and power of turbine rotors. It is relatively simple and computationally fast in meeting the control loop’s accuracy and computational speed requirements.

In general, the generator model chosen for the MCTs’ system is the DFIG system, which is the widespread, basic model for the current fabrication of most WTs [45].

Turbine systems based on the DFIG model in offshore turbines, such as WTs, offer several advantages, including variable speed operation and four-quadrant active and reactive power capabilities. This system also results in lower converter costs and lower energy losses compared to a system based on a fully fed, synchronized generator with a full-ratio converter. Moreover, the generator is robust and requires little maintenance.

Given the extreme similarity of the functional model of the wind generator and the marine current turbine discussed above, the methods for condition monitoring, fault diagnosis, and predictive maintenance of these devices are identical.

Separately, initial research [46] addressed the MCT control problem by considering a linearization of the control of a DFIG system. However, due to the inherent characteristics of offshore currents, such as turbulence, sea swell, and other uncertainties, the initial use of PI-type controllers for subsea turbine speed tracking obtained poor results and low reliability.

The control problem [45] has to be addressed in the context of robust and nonlinear control techniques, and, specifically, work developed using sliding control techniques is of interest.

For DFIG-type turbines, sliding control is quite effective in conversion efficiency, torque swing reduction, and robustness against grid disturbances. The control strategy is as follows:

First, the speed reference (*ω_ref_*) is generated by a maximum power point tracking (MPPT) strategy, followed by calculating an optimal electromagnetic torque using the mechanical equation:

(11) $$T_{em-ref}=T_{m}+f\omega -\alpha \left(\omega -\omega_{ref}\right)+J{\dot{\omega }}_{ref}$$

where *α* is a positive constant, *T_em_* is the electromagnetic torque, *T_m_* is the mechanical torque, *f* is the viscosity coefficient, *J* is the rotor inertia, and *w* is the angular velocity.

Then, the rotor current references are derived to ensure DFIG torque and reactive power convergence and optimum and zero torque.

(12) $$\{\begin{array}{c} \begin{array}{c} I_{qr_{ref}}=-\frac{L_{s}}{pM}\frac{T_{em\_ref}}{\Phi_{sd}}\\ I_{dr_{ref}}=\frac{1}{M}\Phi_{sd} \end{array} \end{array}$$

where *s* and *r* are the rotor and stator indices, *d* and *q* refer to the synchronous reference frame, *V* is the voltage, *I* is the current, *R* is the resistance, *L* and *M* are the self-inductance and mutual inductance, *Φ* is the flux, and *p* is the number of pole pairs.

The following areas are defined:

(13) $$\{\begin{array}{c} S_{1}=I_{dr}-I_{dr\_ref}\\ S_{2}=I_{qr}-I_{qr\_ref} \end{array}$$

Next,

(14) $$\{\begin{array}{c} \begin{array}{c} {\dot{S}}_{1}=\frac{L_{s}}{M^{2}-L_{r}L_{s}}(V_{dr}+R_{r}I_{dr}-\omega_{r}\left(L_{r}I_{qr}+MI_{qs}\right)\\ -\frac{M}{L_{s}}V_{ds}-\frac{MR_{s}}{L_{s}}I_{ds}+\frac{M}{L_{s}}\omega_{s}\left(L_{s}I_{qs}+MI_{qr}\right))-{\dot{I}}_{dr\_ref}\\ {\ddot{S}}_{1}=\varphi_{1}\left(t,x\right)+\gamma_{1}\left(t,x\right)V_{dr}\; \end{array} \end{array}$$

(15) $$\{\begin{array}{c} \begin{array}{c} {\dot{S}}_{2}=\frac{L_{s}}{M^{2}-L_{r}L_{s}}(V_{qr}+R_{r}I_{qr}-\omega_{r}\left(L_{r}I_{dr}+MI_{ds}\right)\\ -\frac{M}{L_{s}}V_{qs}-\frac{MR_{s}}{L_{s}}I_{qs}+\frac{M}{L_{s}}\omega_{s}\left(L_{s}I_{ds}+MI_{dr}\right))-{\dot{I}}_{qr\_ref}\\ {\ddot{S}}_{2}=\varphi_{2}\left(t,x\right)+\gamma_{2}\left(t,x\right)V_{qr}\; \end{array} \end{array}$$

where $\varphi_{1}\left(t,x\right),\varphi_{2}\left(t,x\right),\gamma_{1}\left(t,x\right),\;\mathrm{and}\;\gamma_{2}\left(t,x\right)$ are uncertain functions that satisfy

(16) $$\{\begin{array}{c} \varphi_{1}>0,|\varphi_{1}|>\Phi_{1},\;0<\Gamma_{m1}<\gamma_{1}<\Gamma_{M1}\\ \varphi_{\;2}>0,|\varphi_{2}|>\Phi_{2},\;0<\Gamma_{m2}<\gamma_{2}<\Gamma_{M2} \end{array}$$

The proposed second-order sliding mode controller contains two parts:

(17) $$V_{dr}=u_{1}+u_{2}$$

where

(18) $$\{\begin{array}{c} {\dot{u}}_{1}=-\alpha_{1}sign\left(S_{1}\right)\;\\ u_{2}=-\beta_{1}{|S_{1}|}^{\rho }sign\left(S_{1}\right) \end{array}$$

(19) $$V_{qr}=w_{1}+w_{2}$$

(20) $$\{\begin{array}{c} {\dot{w}}_{1}=-\alpha_{2}sign\left(S_{2}\right)\;\\ w_{2}=-\beta_{2}{|S_{1}|}^{\rho }sign\left(S_{2}\right) \end{array}$$

To ensure convergence, gains are selected as follows:

(21) $$\{\begin{array}{c} \alpha_{1}>\frac{\Phi_{1}}{\Gamma_{mi}}\;\\ \beta_{i}^{2}\geq \frac{4\Phi_{1}}{\Gamma_{mi}{}^{2}}\frac{\Gamma_{Mi}\left(\alpha_{i}+\Phi_{i}\right)}{\Gamma_{mi}\left(\alpha_{i}-\Phi_{i}\right)};i=1,2\;\\ 0<\rho \leq 0.5\; \end{array}$$

Simulations of this rotor speed control strategy versus its reference are shown in [46].

### 4.2. MCT Types

Theoretical studies and experimental projects are being carried out in some countries such as the United Kingdom, France, Italy, Canada, Japan, Russia, Australia, and China. More specifically, two prototypes are being developed with partial funding from the European Commission. The United Kingdom is a world leader in research on obtaining energy from sea currents and waves in various forms (kinetic and potential). In recent years, its government has invested over GBP 60 million and approved programs for developing demonstration facilities and MCT prototypes. They are aware that energy from the sea will soon make up between 15% and 20% of the energy generated in the UK.

The total power of ocean currents is estimated to be about 5 TW [3,38]. However, energy extraction is feasible only in some areas where currents are concentrated near the periphery of the oceans or by straits and passages between islands and other geographical features. Thus, only a part of the total energy can be converted into electrical or other power. Some of the models of MCTs that have been developed [40,45] are presented below.

Researchers and firms have tested different MCT concepts since the beginning of the 21st century. OpenHydro (OpenHydro, Dublin Ireland)was the first prototype tested in real conditions, and a 500 kW turbine was commissioned in September 2011 in France. The DCNS tidal subsidiary is working on a project with this concept to install a 4 MW tidal array in Canada [4].

SeaGen (Simec Atlantis Energy, Edinburgh, United Kingdom) was the world’s first commercial MCT [47]. With a capacity of 1.2 MW, it was commissioned in Northern Ireland’s Strangford Lough in July 2008. The design included two rotors in each structure.

The MeyGen project by SIMEC Atlantis Energy in Scotland, UK, is the world’s biggest, planned MCT farm. It is intended to generate up to 398 MW. MeyGen phase 1 includes AR1500 turbines provided by Atlantis Resources and AH1000 MK1 from Andritz Hydro Hammerfest (Andritz, Vienna, Germany).

Another interesting project is being implemented on Ouessant Island (France). The Sabella project installed a 1 MW tidal turbine grid connected to Ushant Island in 2015.

**Figure 4:**
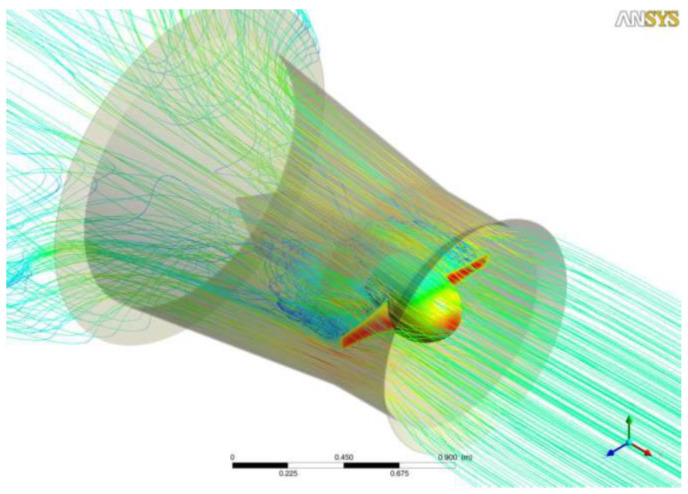
Surge channel to increase the flow speed.

The electric generation capacity of MCTs can be evaluated through computational fluid dynamics (CFD) modeling and simulation [48]. Relative to a free-flow turbine, closed-flow turbines equipped with surge channels (Figure 4) can have three to four times greater efficiency [17].

## 5. Integrated Kinetic Hydro–Wind Power System

**Figure 5:**
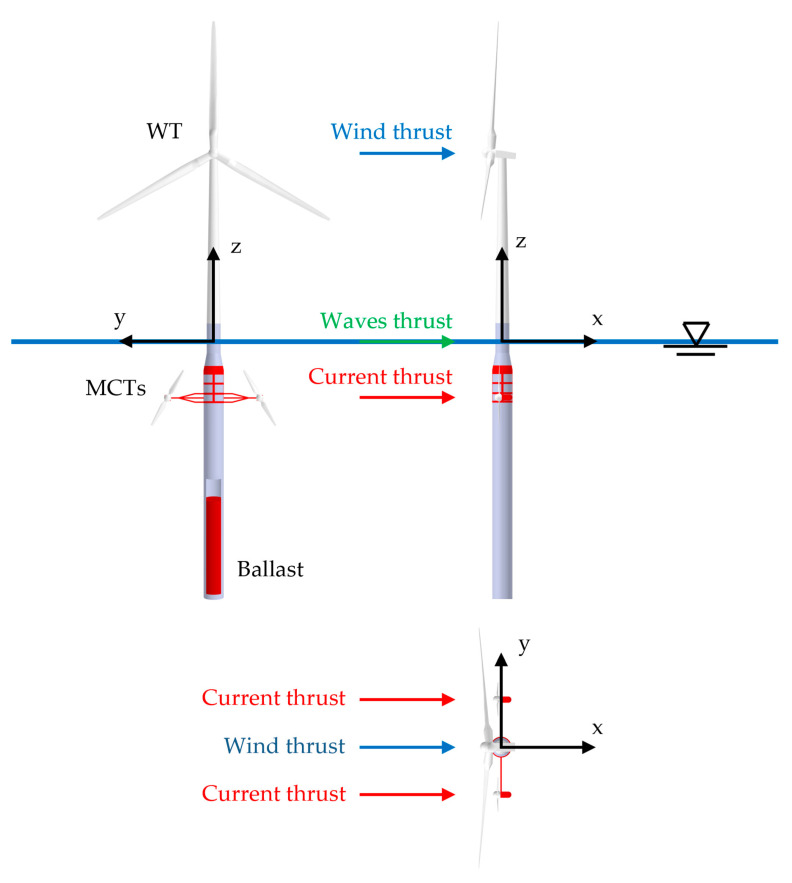
MICHEGER prototype.

**Figure 6:**
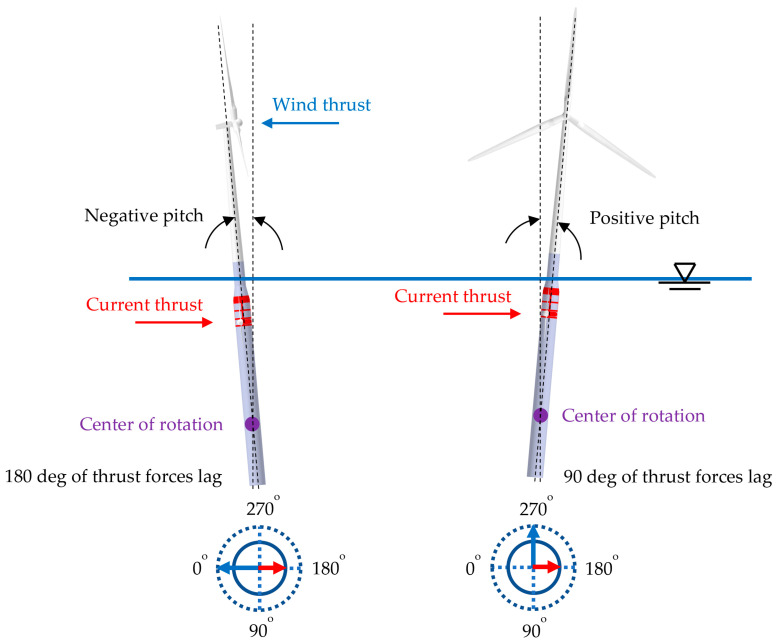
Combinations of force vectors.

The FWT and MCT control problem was discussed in the previous sections, with consideration given to the specific control criteria for each kind of generator. Previous research [7,49,50,51,52,53] proposed an integrated generation system that presents different advantages. The control algorithm considers the interaction of the two integrated generation subsystems (WT and MCT, see Figure 5) to take advantage of the FWT and MCT generation jointly. The possible combinations of the force vectors resulting from the wind and the currents and waves are shown in Figure 6.

In the case of a floating system, stability is the property of the system to recover or maintain the equilibrium position after it is lost due to the forces acting on it. The ideal position is the one of maximum righting that it is not heeled in the axial or the transverse direction, since, in this position, there are fewer structural loads.

**Figure 7:**
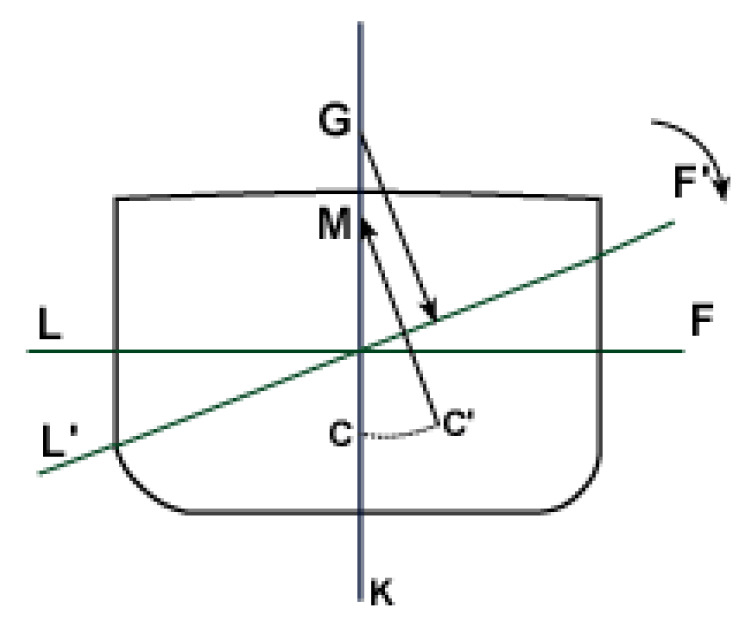
Floating stability.

The stability of the floating system depends on the simultaneous position of its gravity center (CoG), its center of hull or pressure C, and the relative position of both with a third point called metacenter M (Figure 7). When the floating system tilts due to the effect of balance, the shape of the hull changes, and, therefore, its center of hull also varies, originating a pair of forces: one applied downward at the CoG and another applied upward at the center of hull (C′) called transverse stability torque or righting torque, which forces the floating system to stay upright.

In the absence of righting torque, the floating system could flip. This usually happens when the metacenter is below the center of gravity (negative stability). The most critical case of force mismatch is when wind and hydrodynamic forces contribute in a superimposed way to the structural imbalance of the system.

As can be seen in the graph in Figure 5, the main forces acting on the floating system are the wind, the waves, and the ocean currents. Although wind and waves are in phase most of the time, as the latter depends on the former, this is not the case with ocean currents, which do not necessarily depend on the latter. This phase difference can range up to ±180°, so, eventually, the acting forces may be superimposed across or opposing to a greater or lesser degree.

In critical operating conditions due to adverse weather conditions in the marine environment, where it is necessary to prioritize the objective of safety, the authors propose the eventual possibility of using part of the energy generated to increase the drive level of the control system.

From the basic modeling considerations made in Section 3, and for the case of an FWT/MCT generation system [17], it is proposed to use precisely the actuation force component $\tau_{j}^{A}$ to use a reversible actuation of the MTCs to contribute to the structural stability of the floating device in adverse, critical situations.

The idea is to take advantage of reversible generator/motor operation of the MCTs to make them work as actuators that contribute to the structural stabilization of the floating system. The idea is for the MCTs to generate counteracting and cooperating forces to those that are superimposed against the structural stability of the system.

**Figure 8:**
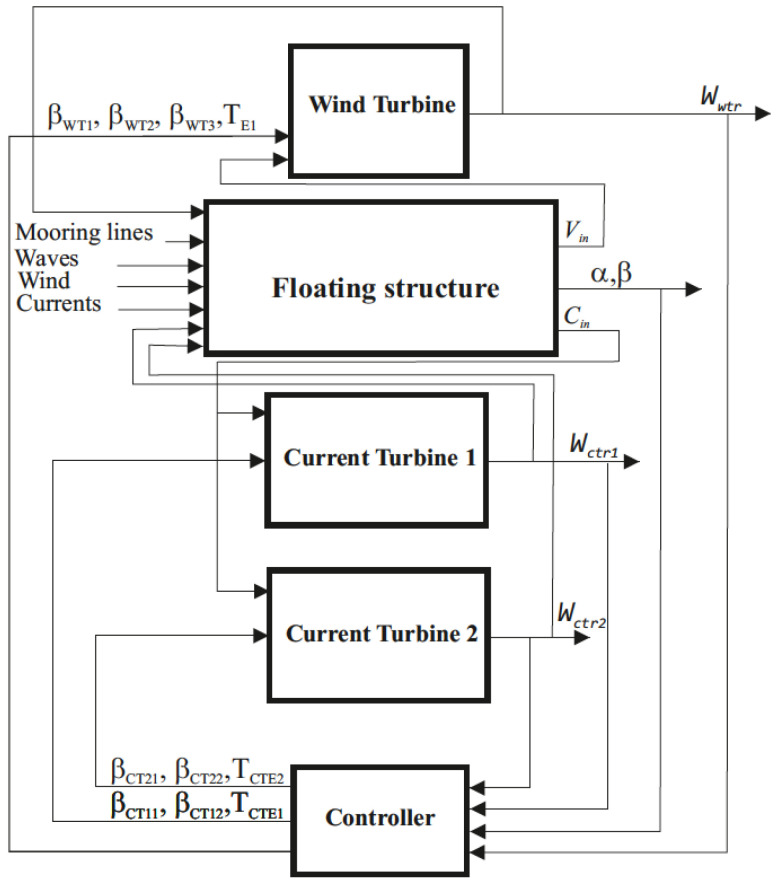
Control proposal of the integrated system.

Taking into account the considerations made, a control proposal on the integrated system is presented in Figure 8, where the control variables are the torques of the wind turbine *T_wt_* and the current turbines *T_ct1_* and *T_ct2_*; the blade angles of the wind turbine *β_wt1_*, *β_wt2_*, and *β_wt3_* and of the two MCTs, *β_ct11_*, *β_ct12_*, *β_ct21_*, and *β_ct22_* controlled individually.

The output variables are the pitch angle (*α*) and roll angle (*β*) to the CoB of the structure; the angular velocities of the rotors *w_wt_*, *w_ct1_*, and *w_ct2_*, and the generated powers *P_wt_*, *P_ct1_*, and *P_ct2_*. *V_in_* and *C_in_* represent the interaction of the FWT and the two MCTs with the floating structure, respectively.

## 6. Requirements in the Supervision Layer

The implementation of generation plants is conditioned by previous studies based on the economic viability and sustainability of the project in the long term. From these studies, it can be seen that not only does the initial investment need to be considered, but also the operating expenses. Of the latter, the operating and maintenance costs of the facilities form a special and sometimes decisive part. To achieve a reduction in these costs, especially in marine installations, integrated condition monitoring and fault diagnosis systems are required for intelligent management of the process maintenance, a requirement that is demanded by European insurance companies [54].

In order to make an offshore wind farm profitable, stoppages due to breakdowns must be avoided as much as possible; therefore, it is necessary to develop failure models for wind turbines and optimal planning of maintenance operations. To do this, once a failure occurs, or is anticipated through the intelligent condition monitoring systems, algorithms are needed that analyze all the variables based on operating experience, failure data, logistics information, availability of access, human resources, spare parts, etc., along with the weather forecast [55].

Although fault diagnosis includes the objectives of fault detection, isolation, and analysis, allowing the fault to occur can result in serious financial losses. The locking of a bearing can lead to a catastrophic failure. This is especially important in the specific case of wind generators. The philosophy of condition monitoring techniques, extended to numerous industrial processes, is basically predictive to the extent that it pursues the objective of not allowing the failure to occur. The aim is to detect symptoms in early phases, allowing prediction of the occurrence of the failure within an appropriate time interval, so it is possible to undertake the appropriate maintenance tasks in the best conditions. The greatest experiences with condition monitoring systems have been gained, for example, in the chemical and paper industries. These have elements in common, i.e., their devices work in stationary conditions. However, wind generators suffer from stochastic loads, which make it extremely difficult to analyze the measured data. The latter problem is increased in the marine environment. This is a challenge for the large-scale development of fault diagnosis systems in the offshore wind industry.

The previous sections focused on the technologies necessary to generate energy from the different sources that the seas and oceans offer us. From the point of view of its future viability, there are some aspects that cannot be neglected; supervision and predictive diagnosis of faults are vital from the point of view of maintenance and, therefore, of economic viability.

In offshore wind farms, it is essential to have a supervision system that monitors the main generation indicators. Modern supervision systems already include certain diagnostic features, although, in the generation systems we are dealing with, they are essential for more reasons. In the first place, the ocean is a highly corrosive medium, and, although the design and the materials used take this into account, any failure can cause degradation of the elements that make up the plant faster than it would on land. Secondly, the maintenance operation, both corrective and preventive, is more complex and expensive than onshore. This is due to the marine transport of materials and people and, in addition, to the need to include meteorology as a fundamental factor in the planning of these activities. Finally, a critical failure in one of the generators of an offshore plant can affect the rest of the generators, causing serious economic damage.

The diagnostic systems that are used in a traditional way are necessary but not sufficient, since the detection of a fault triggers a corrective maintenance order that may not be carried out due to weather conditions. From this point of view, and without prejudice against the use of classic fault diagnosis techniques, the systems used in the marine environment must especially be based on predictive condition monitoring techniques [55,56,57].

**Figure 9:**
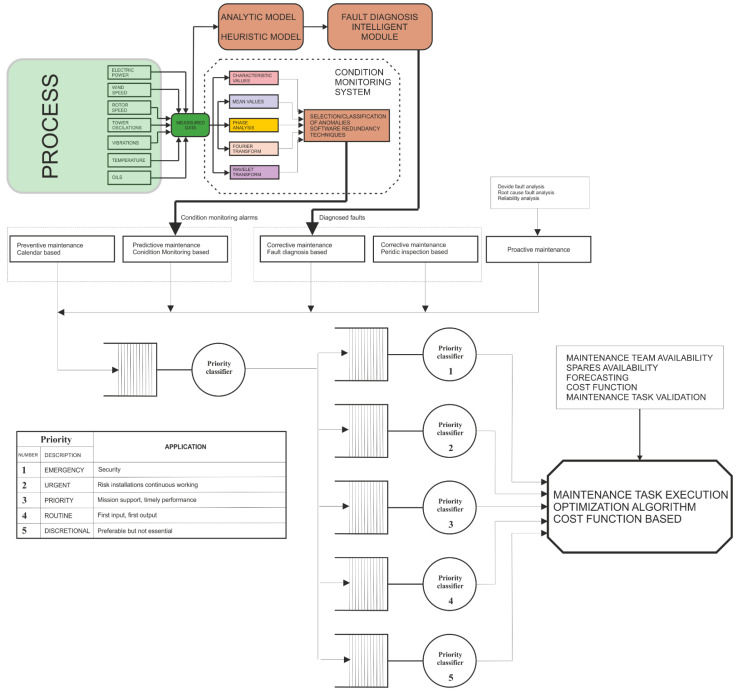
Block diagram of the proposed condition monitoring system.

Figure 9 shows a condition monitoring module including the measured variables, the applied diagnosis techniques, and the integration with a complete maintenance system. The results of the diagnostics are used as inputs to maintenance blocks that issue the corresponding maintenance action orders, characterized by a certain priority level and affected by a cost function.

In this type of intelligent condition monitoring system, a diverse set of diagnostic techniques is often used redundantly. For instance, in an offshore wind turbine, the predictive subsystem should integrate:

- Trend analysis techniques;
- Vibration analysis techniques;
- Ultrasonic analysis techniques;
- Thermographic analysis techniques;
- Oil analysis techniques.

The predictive maintenance system can be complemented by a corrective subsystem that includes:

- Analytical diagnostic models;
- Heuristic diagnostic models;
- Fault tree models.

In wind turbines, status monitoring methods range from very general diagnosis to techniques that are focused on mobile elements or structural elements [49,56]. These techniques can be extrapolated to most generation systems, although they require adaptation of the specific devices in each case.

In the case of general diagnosis, the power performance curve is usually used with respect to the renewable resource used. In the case of wind turbines, the wind power curve tells us if the wind turbine is working as expected or if there is a problem. Deviations from what is expected can be analyzed and a degradation trajectory of the entire system studied, although they do not point to which particular subsystem is degrading.

### 6.1. Moving Elements

Vibration analysis, in its different modalities, continues to be the most widely used technique for monitoring the state of turbines and is especially used for rotary systems that include shafts and bearings. The algorithms used in machines can be consulted in several previous studies [57]. Similar techniques can also be used to monitor the structural behavior of the system at lower frequencies.

More advanced methods are based on spectral analysis techniques to detect frequencies that correspond to periodic excitations caused by specific faults, such as pitting on the outer face of bearings [57]. Additionally, an efficient technique for gearboxes and bearings is the envelope curve analysis, which focuses on the analysis of high-frequency modulation due to low-frequency excitations produced by certain faults [58].

Another technique that is used redundantly in the analysis of rotating machines is oil analysis. Although, in the past, this technique was used offline, modern online sensors have become cheap enough to be competitive and can be installed in lubrication systems without much problem.

Classic diagnostic techniques with electrical models are also used when the model is available. In this instance, the variations in resistance and inductance reflect the degradation of the components.

### 6.2. Structural Elements

The modifications that can occur on metallic structures produce pressure waves that can be analyzed in different ways. In [59], it was shown that acoustic emission analysis techniques can determine failures before vibration analysis. The main difference between acoustic sensors and vibration sensors is that they are attached to the component to be measured in order to detect displacement, while acoustic sensors are mounted with flexible glue and measure the sound directly.

On the other hand, ultrasonic and radiological testing techniques [60] have been used in the world of wind generation since the beginning to look for structural problems. A review of these ultrasonic methods can be found in [61,62].

### 6.3. Trend Analysis

Trend analysis is a quantitative technique to identify potentially hazardous conditions based on past empirical data [63].

The study of the reliability and safety of a component or system, in the sense of its most complete quantitative knowledge, should focus on the evaluation of a reliability function (or, equivalently, a risk function). The reliability of a system is a probability function. More specifically, it is the probability of giving adequate performance under specified conditions up to a given time. Usually, this reliability function is a function of time and some other parameters. For example, a widely used wear-to-failure model is the Weibull model. More generally, sometimes the reliability function parameters are themselves functions of other variables, such as pressure and temperature.

Determining the reliability distribution and estimating its parameters can be very difficult, expensive, and, in some cases, intractable. Trend analysis is an alternative or complementary approach. Specifically, it is known that the values of certain variables directly impact the reliability of the system or component, even though the exact quantitative relationship or risk has not been determined. Measurable variables that directly affect the reliability of the system or component are sampled over time.

Variable values are examined to see if there is a pattern of deviation over time (i.e., a trend) from acceptable performance limits. In this way, one may be able to predict future values of the parameters or at least estimate the long-term range of the values of these influential variables. In turn, if these parameters tend towards dangerous or unacceptable levels, the potential problem can be identified before high-risk situations occur [55].

## 7. Conclusions

Prototypes such as the hybrid FWT/MCT generator proposed in this work can contribute to facilitating the implementations of offshore wind turbines. From the point of view of control engineering, research tasks relating to this type of project should focus on developing advanced control algorithms, the initial objective of which is preserving their structural stability. The goal is to avoid overloads in harsh operating conditions, guaranteeing their useful life extension.

In parallel, applying and developing monitoring techniques based on predictive condition monitoring are essential for avoiding catastrophic failures. Moreover, these supervision techniques increase the energy efficiency of MCTs, which has been identified as their weakest point.

In the author’s opinion, the proposal to use hybrid FWTs/MCTs as marine current generators goes in the direction of increasing the economic viability of investments. These foundations promote the definitive start-up of marine generators, which have a promising future.

The level of offshore energy activity is still very low compared to onshore energy generation, so exports and imports are not significant. The increased use of sustainable energy will also have significant social consequences. The widespread use of renewable energy systems contributes to the change of the productive model, as is evidenced by the case of wind power generation. Other social impacts will come from the change in international relations as certain nations end their dependence on third parties for energy and the expected improvements in health due to not being exposed to the emissions associated with fossil fuels.

Wind energy has great potential to contribute to the EU target of obtaining 45% of energy consumed from renewable energy sources by 2030. To achieve higher turbine availability and reduce the cost of wind energy, developing new diagnostic methods to reduce maintenance costs and improve reliability is an essential element to consider. This research is especially relevant in the case of offshore wind generation, where the difficulty of maintenance operations considerably increases these costs.

In onshore wind farms, the annual operation and maintenance cost is estimated to be between 3% and 5% of the total installation cost. A fundamental objective is to reduce these costs by providing predictive maintenance tools to improve the planning of maintenance operations. In this regard, a model of intelligent integration of the tasks of supervision, diagnosis, and predictive maintenance is proposed in this work.

As future work, studies should be dedicated to analyzing the feasibility and functional capacity of the different types of marine generator that can be integrated into floating platforms and foster the development of an integrated control, the objective of which is to guarantee the structural stability of the system and reach optimal levels in generation capacity.
